# Epidemiological and clinical impact of hepatitis E virus coinfection in chronic hepatitis B infected patients in Hebei, China

**DOI:** 10.3389/fmicb.2025.1638614

**Published:** 2025-08-11

**Authors:** Yuzhu Shi, Yuchen Xie, Ying Chen, Li Yang, Huixia Gao, Yun Guo, Fumin Feng, Jianhua Lu, Erhei Dai

**Affiliations:** ^1^School of Public Health, North China University of Science and Technology, Tangshan, China; ^2^Hebei Key Laboratory of Immune Mechanism of Major Infectious Diseases and New Technology of Diagnosis and Treatment, The Fifth Hospital of Shijiazhuang, North China University of Science and Technology, Shijiazhuang, Hebei, China

**Keywords:** hepatitis B virus, hepatitis E virus, coinfection, liver injury, epidemiology

## Abstract

**Introduction:**

Hepatitis B virus (HBV) infection poses a major global public health challenge. Recent studies have highlighted the clinical implications of coinfection with the hepatitis E virus (HEV) in HBV-infected individuals, as this dual infection is associated with exacerbated disease severity. However, epidemiological data on HBV/HEV coinfection in the Hebei region are scarce, necessitating further investigation.

**Methods:**

We conducted a multicenter cross-sectional study for infectious diseases across six tertiary hospitals. Serum samples were screened for anti-HEV IgM and IgG antibodies by using an automated chemiluminescent immunoassay. Samples positive for anti-HEV antibodies were further subjected to HEV RNA detection using the reverse transcription polymerase chain reaction (RT-PCR). The seroprevalence of anti-HEV antibodies was 18.70% (582/3,113).

**Results:**

Among these, 562 cases were positive for anti-HEV IgG, 4 were positive for anti-HEV IgM, and 16 were positive for both anti-HEV IgG and IgM. HEV RNA was detected in 16 (2.75%; 582) anti-HEV-seropositive individuals. A strong positive correlation was observed between anti-HEV seroprevalence and advancing age [*R*^2^ = 0.966 in the liver cirrhosis (LC) group, *R*^2^ = 0.774 in the hepatocellular carcinoma (HCC) group, and *R*^2^ = 0.508 in the chronic hepatitis B (CHB) group]. Multivariate analysis confirmed that older age was an independent risk factor for anti-HEV seropositivity (OR = 1.03, 95% CI: 1.02–1.04, *P* < 0.001). HBV mono-infection patients were significantly younger than those with HBV and acute HEV coinfection patients or HBV with previous HEV infection patients (53.0 vs. 58.5 vs. 58.0 years, *P* < 0.001). Additionally, LC and HCC were more prevalent in the HBV with previous HEV infection than in HBV mono-infection (65.98% vs. 77.27%, *P* < 0.001). Compared to HBV mono-infection patients, the activated partial thromboplastin time was significantly prolonged in both HBV and HEV acute coinfection patients and in those with HBV and previous HEV infection (32.30 s vs. 35.65 s vs. 34.46 s, *P* < 0.05).

**Discussion:**

Our findings demonstrated an 18.70% seroprevalence of anti-HEV antibodies among chronically HBV-infected patients in the Hebei Province, with a significantly higher risk of coinfection in older individuals. HBV/HEV coinfection may aggravate liver injury and impair coagulation. These results provide valuable insights into the epidemiology and clinical consequences of HBV/HEV coinfection in this region.

## 1 Introduction

Hepatitis virus (HBV) infection is a major global health burden, with progression from chronic hepatitis B (CHB) to liver cirrhosis (LC) and subsequent development of hepatocellular carcinoma (HCC) (Lai and Yuen, 2007). The World Health Organization (WHO) (2022) estimated 254 million chronic HBV carriers worldwide, contributing to 1.2 and 1.1 million new infections and annual deaths, respectively, from LC and HCC complications. In China, the prevalence of HBsAg in the general population is 5.86%, corresponding with approximately 75 million cases of chronic HBV infection (Hui et al., 2024).

Hepatitis E virus (HEV), a 30–40 nm single-stranded positive-sense RNA virus of the *Paslahepevirus* genus, has eight distinct genotypes with differential transmission patterns (Liu et al., 2021; Tam et al., 1991). HEV can be transmitted by both horizontal and vertical routes, with vertical propagation being the primary model of transmission, particularly though fecal-oral transmission (Nagashima et al., 2017). HEV-1 and HEV-2 are waterborne viruses that are prevalent in developing countries with poor sanitation. Conversely, HEV-3 and HEV-4 are widespread in both developed and developing countries and are primarily transmitted through animal sources (Khuroo et al., 2016; Nelson et al., 2019; Wang and Meng, 2021). Notably, HEV-4 is predominant in China and is transmitted through animal sources, with pigs and other animals serving as primary sources of infection (Tang et al., 2019; Tang et al., 2023; Wang et al., 2016).

Globally, the annual incidence of HEV infection exceeds 20 million, with 44,000 fatalities (Chinese Society of Hepatology, and Chinese Medical Association, 2022). China exhibits particularly high endemicity, with a 23.46% anti-HEV seroprevalence population (Jia et al., 2014). A 2022 review reported that the HEV IgM antibody positivity rate in Hebei Province was the highest in China (3.13%) (Cao et al., 2023). Serological screening of livestock in this intensive farming region revealed a high anti-HEV antibody seroprevalence across species, with 90.4%, 12.2%, and 10.9% of pigs (HEV-4), donkeys (HEV-3), and rabbits (HEV-3), respectively, testing positive, establishing substantial zoonotic transmission risks (Geng et al., 2019; Rui et al., 2020; Zhang et al., 2023). These epidemiological and zoonotic factors substantially increase the population’s exposure to HEV, thereby raising the likelihood of HBV/HEV coinfection, particularly in high-endemic regions.

Clinical synergism between HBV/HEV coinfection exacerbates hepatic outcomes, particularly in patients with LC or HCC who demonstrate heightened susceptibility to acute/subacute liver failure with several-fold mortality risk elevation (Nasir and Wu, 2020; Ramachandran et al., 2004; Zhang et al., 2017). Moreover, emerging evidence has identified HEV coinfection as an independent risk factor of accelerated HBV-related disease progression (Chen et al., 2016; Tseng et al., 2020).

Despite the growing recognition of the clinical importance of HBV/HEV coinfection, there remains limited understanding of its epidemiology and clinical outcomes, especially in Hebei, where data are scarce. This study aimed to systematically investigate the prevalence, clinical profiles, and risk determinants of CHB/HEV coinfection patients in Hebei. These findings may inform targeted screening protocols and personalized therapeutic strategies for vulnerable populations.

## 2 Materials and methods

### 2.1 Study design and sample selection

This multicenter, cross-sectional study was conducted between June 2023 and March 2024 at six hospitals in the Hebei Province, China: Shijiazhuang Fifth Hospital, Baoding People’s Hospital, Tangshan Seventh Hospital, Handan Infectious Disease Hospital, Cangzhou Third Hospital, and Zhangjiakou Infectious Disease Hospital. The study protocol was approved by the Medical Ethics Committee of Shijiazhuang Fifth Hospital (approval number 2022-013).

A total of 3,113 HBsAg-positive patients with chronic HBV-infected infection were enrolled. Inclusion criteria were as follows: (1) chronic HBV infection with HBsAg positivity for over 6 months and current hospitalization; and (2) age ≥ 18 years, regardless of gender. The exclusion criteria were as follows: (1) HCV infection; (2) pregnancy or lactation; (3) insufficient serum sample volume; and (4) incomplete clinical data.

Using the 2023 Expert Consensus on the Hospital Screening Management Process for Hepatitis E in China (Chinese Consortium for the Study of Hepatitis E (CCSHE), 2023), we classified 3,113 patients into three groups based on their infection status:

- HBV mono: HBV mono-infection group (anti-HEV IgM and IgG negative);
- HBV/HEV acute: HBV and acute HEV coinfection group (anti-HEV IgM positive or HEV RNA positive); and
- HBV/HEV previous: HBV with previous HEV infection group (anti-HEV IgG positive and IgM negative).

### 2.2 Data collection

Personal demographic information, clinical data, laboratory results, and imaging results were obtained from the hospital’s electronic medical record system.

Personal information included the patient’s sex, age, family history of HBV infection, time since onset, clinical diagnosis (CHB, LC, and HCC), residential area, occupation, and antiviral medication.

Laboratory tests included routine blood tests, comprising white blood cells, hemoglobin (Hb), and platelet count (PLT); liver and kidney function tests, including alanine aminotransferase (ALT), aspartate aminotransferase (AST), gamma-glutamyl transferase, albumin, globulin, total bilirubin (TBIL), and direct bilirubin; coagulation function tests, such as prothrombin time (PT), international normalized ratio (INR), prothrombin time activity (PTA), activated partial thromboplastin time (APTT), fibrinogen, and thrombin time; and HBV serological markers, including serum HBsAg, hepatitis B e antigen (HBeAg), hepatitis B e antibody (HBeAb), and serum HBV DNA.

Imaging studies included gastroscopy, abdominal ultrasound, abdominal computed tomography (CT), and magnetic resonance imaging.

### 2.3 Serological and virological assays

Serum samples were separated by centrifugation at 3,000 rpm for 15 min and immediately stored at −80°C. Except for Shijiazhuang samples, all samples were transported to the central laboratory of the Fifth Hospital of Shijiazhuang for uniform testing through a cold chain system (temperature monitored at −20°C).

Anti-HEV IgG/IgM detection serum anti-HEV IgG and IgM antibodies were detected using HEV IgG and HEV IgM antibody test kits based on the magnetic particle chemiluminescence method. Assays were conducted using a fully automated magnetic particle chemiluminescence analyzer (AutoLoumo A2000), with test kits and equipment provided by AutoBio Diagnostics Co., Ltd. (Zhengzhou, China). All procedures were carried out using the manufacturer’s instructions, with S/CO ≥ 1 considered a positive result.

The serum HEV RNA was extracted using an automated bead-based nucleic acid extraction system (Daan Gene, Guangzhou, China). The extraction process strictly adhered to the instructions for the Daan Gene Viral Nucleic Acid Purification Kit. Serum HEV RNA was detected using an HEV nucleic acid detection kit (PCR-fluorescent probe method) (ACON Bio Co., Ltd., China) and real-time fluorescence quantitative PCR (Applied Biosystems, United States) as the instrument used. The reverse transcription polymerase chain reaction (RT-PCR) mixture consisted of 20 μl, including 18 μl RT-PCR mixture, 1.4 μl enzyme mixture, and 0.6 μl HEV fluorescent probe. In the initial assay, samples with Ct values ≤ 38.0 were considered positive, while samples with Ct values ≥ 40 were classified as negative. A Ct value range between 38 and 40 was defined as the “gray zone,” where low-concentration samples showed lower precision. Samples with Ct values above 38 were re-tested to ensure diagnostic accuracy.

### 2.4 Statistical methods

Quantitative data (non-normal distribution): Expressed as median and interquartile range (IQR), with group comparisons performed using the Mann–Whitney *U* test or Kruskal–Wallis *H* test.

Categorical data: Presented as percentages (%), and differences between groups were evaluated using the Chi-square test or Fisher-Freeman-Halton exact test.

*Post hoc* pairwise comparisons: In cases of statistically significant differences among three groups, pairwise comparisons were adjusted using the Bonferroni method.

Linear regression analysis: Used to fit the data, and the goodness of fit was assessed by the *R*^2^ value.

Binary logistic regression: Analyzed factors influencing outcomes, with results expressed as odds ratios (ORs) and 95% confidence intervals (CIs).

Statistical analysis was performed using SPSS software, and pie charts and bar graphs were generated using GraphPad Prism 9.0 (San Diego, CA, United States). A *P*-value of <0.05 was considered statistically significant.

## 3 Results

### 3.1 Seroprevalence of hepatitis E virus coinfection in chronic HBV-infected patients

**FIGURE 1:**
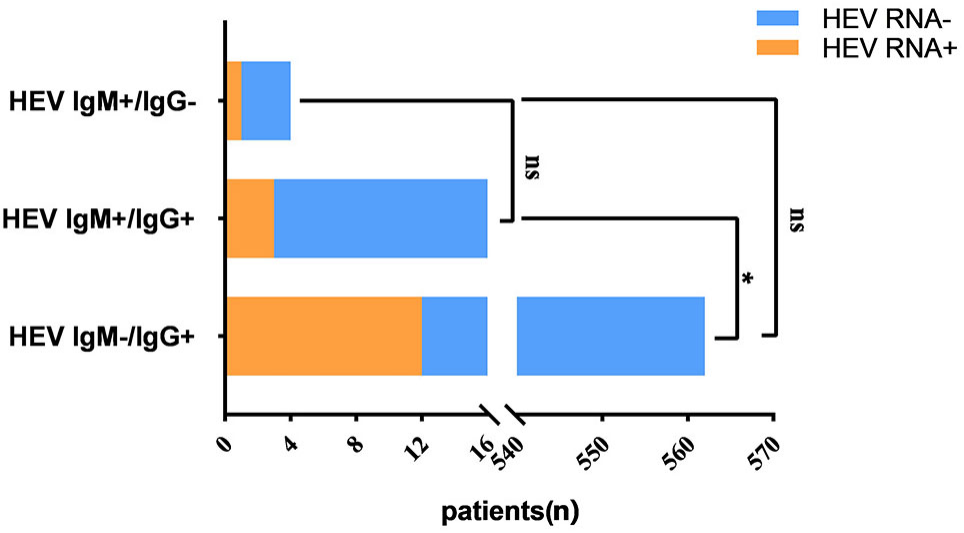
Anti-HEV seroprevalence and HEV RNA positivity rates in chronic HBV-infected patients. Group comparisons were performed using Fisher’s exact test. ns = non-significant (*P* > 0.05); *P < 0.05.

Of the 3,113 chronic HBV inpatients screened in the Hebei Province, HEV seropositivity was detected in 582 cases, yielding an overall anti-HEV prevalence of 18.7% (IgG+, 18.57%; IgM+, 0.64%; dual positivity, 0.51%). Among the 582 seropositive individuals, 16 (2.75%) had detectable HEV RNA levels. The stratified analysis revealed significantly elevated RNA detection rates in specific subgroups: 25.0% in IgM+ patients and 18.75% in IgM+/IgG+ dual-positive cases, compared to 2.14% in IgG+ mono-reactive individuals (*P* = 0.006) (Figure 1).

### 3.2 Geographic heterogeneity of HEV seroprevalence

Among the six regions in Hebei Province, anti-HEV IgG positivity rates varied significantly (χ^2^ = 59.289, *P* < 0.001). Zhangjiakou had the highest rate (35.39%), followed by Handan (24.49%), Tangshan (19.62%), Cangzhou (17.87%), Shijiazhuang (14.82%), and Baoding (13.46%). Pairwise comparisons showed that: (1) Zhangjiakou exhibited a significantly higher anti-HEV IgG positivity rate than Handan (*P* = 0.005) and all the other regions (*P* < 0.001); and (2) Handan had a significantly higher rate than did Baoding and Shijiazhuang (all *P* < 0.001).

No significant geographic variation in anti-HEV IgM seroprevalence was observed (*P* > 0.05). The rates ranged from 0.48% in Baoding to 1.01% in Handan, with comparable values in other regions (Table 1).

**TABLE 1:** Prevalence of anti-HEV IgM and IgG in HBsAg-positive patients in different regions.

Region	Case (*n*)	HEV IgG positivity case, *n*	HEV IgG positivity rate, %	HEV IgM positivity case, *n*	HEV IgM positivity rate, %
Zhangjiakou	178	63	35.39	1	0.56
Handan	592	145	24.49	6	1.01
Tangshan	367	72	19.62	2	0.54
Cangzhou	263	47	17.87	2	0.76
Shijiazhuang	1,505	223	14.82	8	0.53
Baoding	208	28	13.46	1	0.48
*P*		<0.001^a^		>0.05^b^	

^a^Used the Chi-square test.

^b^Used the Fisher-Freeman-Halton exact test.

### 3.3 Univariate and multivariate analysis of factors affecting serum anti-HEV seropositivity rates

This study analyzed factors influencing anti-HEV positivity in 582 patients. Univariate analysis revealed significant differences between the two groups in terms of age, clinical diagnosis, residential area, occupation, PLT, ALT, AST, TBIL, HBsAg, HBeAg, and HBV DNA levels (*P* < 0.05). The results are summarized in Table 2.

**TABLE 2:** Univariate analysis of factors affecting anti-HEV seropositivity in HBsAg-positive patients.

Variables	Anti-HEV negative (*n* = 2,531)	Anti-HEV positive (*n* = 582)	Statistic	*P*
Sex, *n* (%)			χ^2^ = 0.11	0.736
Male	1,765 (69.74)	410 (70.45)		
Female	766 (30.26)	172 (29.55)		
Age (years)	53.00 (43.00, 61.00)	58.00 (52.00, 64.00)	*Z* = −8.63	<0.001
Residence area			χ^2^ = 6.58	0.010
Rural	1,899 (75.03)	466 (80.07)		
Urban	632 (24.97)	116 (19.93)		
Occupation			χ^2^ = 22.77	<0.001
Farmer	1,739 (68.71)	439 (75.43)		
Worker	201 (7.94)	19 (3.26)		
Employee	325 (12.84)	81 (13.92)		
Others	266 (10.51)	43 (7.39)		
Diagnosis, *n* (%)			χ^2^ = 30.60	<0.001
CHB	861 (34.02)	136 (23.37)		
LC	1,099 (43.42)	267 (45.88)		
HCC	571 (22.56)	179 (30.76)		
WBC (10 × 9/L)	4.55 (3.40, 6.05)	4.50 (3.30, 5.96)	*Z* = −1.02	0.310
Hb (g/L)	134.00 (113.00, 150.00)	131.00 (112.00, 147.75)	*Z* = −1.88	0.060
PLT (× 10^9^/L)	131.00 (76.00, 192.50)	118.00 (66.00, 176.75)	*Z* = −3.49	<0.001
ALT (U/L)	34.00 (21.13, 69.65)	29.48 (19.91, 48.63)	*Z* = −4.39	<0.001
AST (U/L)	34.20 (23.00, 66.50)	32.00 (22.00, 57.00)	*Z* = −2.11	0.035
GGT (U/L)	38.00 (21.00, 85.25)	37.20 (21.60, 81.35)	*Z* = −0.22	0.823
ALB (g/L)	39.60 (33.60, 43.90)	38.80 (32.92, 43.31)	*Z* = −1.30	0.193
GLB (μmol/L)	29.00 (25.40, 33.00)	29.40 (25.70, 33.60)	*Z* = −1.59	0.111
TBIL (μmol/L)	20.60 (13.70, 34.90)	21.66 (14.46, 38.60)	*Z* = −2.00	0.045
DBIL (μmol/L)	7.10 (4.30, 14.20)	7.23 (4.37, 15.47)	*Z* = −0.75	0.455
HBsAg (IU/ml)	275.89 (125.78, 1,443.16)	250.10 (94.09, 725.83)	*Z* = −4.53	<0.001
HBeAg, *n* (%)			χ^2^ = 16.93	<0.001
Negative	1,621 (64.05)	425 (73.02)		
Positive	910 (35.95)	157 (26.98)		
HBV DNA (IU/ml)	31.70 (19.90, 50,470.00)	19.90 (19.90, 882.70)	*Z* = −5.14	<0.001

Quantitative data were analyzed using the Mann–Whitney *U* test, and qualitative data were analyzed using the Chi-square test.

Multivariate analysis demonstrated a significant increase of 3% in the risk of HEV positivity for each 1-year increase in age (OR = 1.03, *P* < 0.001). Workers exhibited a significantly reduced risk of HEV positivity compared with farmers (OR = 0.44, *P* = 0.001). Other groups (students, individual workers, and retirees) exhibited a significantly reduced risk of HEV positivity compared to farmers (OR = 0.52, *P* < 0.001) (Table 3). The Hosmer–Lemeshow goodness-of-fit test revealed no significant deviation between the predicted and observed outcomes (χ^2^ = 12.321, df = 8, *P* = 0.137).

**TABLE 3:** Multivariate analysis of factors affecting anti-HEV seropositivity in HBsAg-positive patients.

Variables	Multivariable model				
	β	SE	Wald	*P*	OR (95% CI)
Age (years)	0.030	0.005	41.034	<0.001	1.03 (1.02 ∼ 1.04)
**Diagnosis, *n* (%)**					
CHB					1.00 (Reference)
LC	0.122	0.137	0.788	0.375	1.13 (0.86 ∼ 1.48)
HCC	0.228	0.150	2.295	0.130	1.26 (0.94 ∼ 1.69)
**Residence area**					
Rural					1.00 (Reference)
Urban	−0.125	0.118	1.121	0.290	0.88 (0.70 ∼ 1.12)
**Occupation**					
Farmer					1.00 (Reference)
Worker	−0.822	0.256	10.285	0.001	0.44 (0.27∼ 0.73)
Employee	0.199	0.146	1.874	0.171	1.22 (0.92 ∼ 1.62)
Others	−0.657	0.180	13.310	<0.001	0.52(0.36 ∼ 0.74)
PLT (10*9/L)	0.000	0.001	0.258	0.612	1.00 (0.99 ∼ 1.00)
ALT (U/L)	0.000	0.000	0.084	0.772	1.00 (0.99 ∼ 1.00)
AST (U/L)	0.000	0.001	0.386	0.534	1.00 (0.99 ∼ 1.00)
TBIL (μmol/L)	0.000	0.001	0.277	0.599	1.00 (0.99 ∼ 1.00)
HBsAg (IU/L)	0.000	0.000	3.521	0.061	1.00 (1.00 ∼ 1.00)
**HBeAg positive**					
Negative					
Positive	−0.177	0.112	2.503	0.114	0.84 (0.67 ∼ 1.04)
HBV DNA (IU/L)	0.000	0.000	0.195	0.659	1.00 (1.00 ∼ 1.00)

OR, odds ratio; CI, confidence interval.

### 3.4 Age-stratified seroprevalence patterns across liver disease phenotypes

**FIGURE 2:**
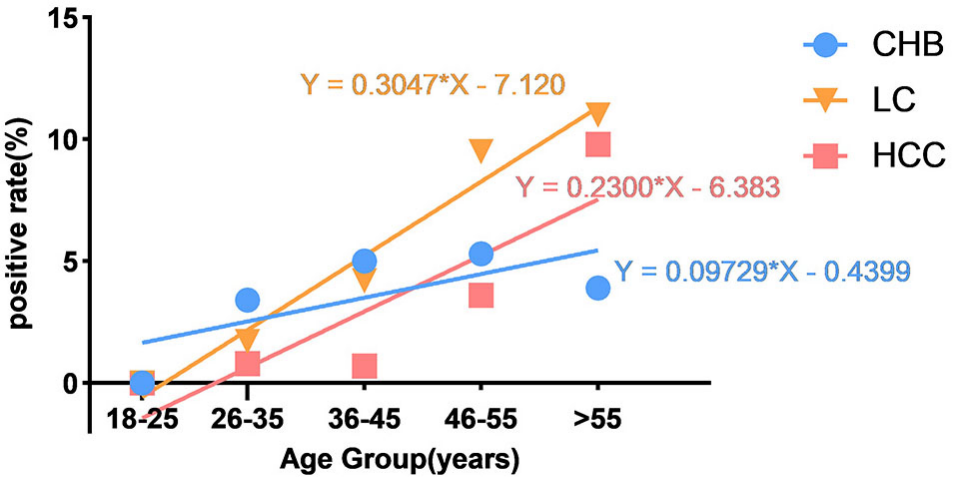
Age-stratified trend analysis of total anti-HEV seroprevalence in chronic HBV-infected patients. The trend lines illustrate the linear relationship between total anti-HEV seropositivity (IgG or IgM positive) and age for each clinical subtype (LC group *R*^2^ = 0.966, HCC group *R*^2^ = 0.774, and CHB group *R*^2^ = 0.508). All data analyses were conducted using a simple linear regression model, stratified by clinical diagnosis.

Chronic hepatitis B group: CHB infection without evidence of LC or HCC. LC group: compensated or decompensated cirrhosis without HCC. HCC group: radiologically/histologically confirmed HCC, including those with concurrent LC. Stratified analysis by clinical diagnosis (CHB, LC, and HCC) and age quintiles (18–25, 26–35, 36–45, 46–55, and >55 years) revealed significant disease-stage-specific anti-HEV seroprevalence gradients (*χ*^2^ = 30.602, *P* < 0.001). The prevalence hierarchy progressed from 13.6% (CHB) to 19.5% (LC) and 23.9% (HCC), with Bonferroni-adjusted pairwise comparisons confirming inter-group differences (*P* < 0.05). Seropositivity rates increased with age in all groups, with a steeper rise in LC and HCC patients compared to those with CHB. The *R*^2^ values were 0.5079 (CHB), 0.9653 (LC), and 0.7735 (HCC), indicating strong model fit for LC and HCC (Figure 2).

### 3.5 Demographic characteristics of patients with different HBV/HEV coinfection states

**FIGURE 3:**
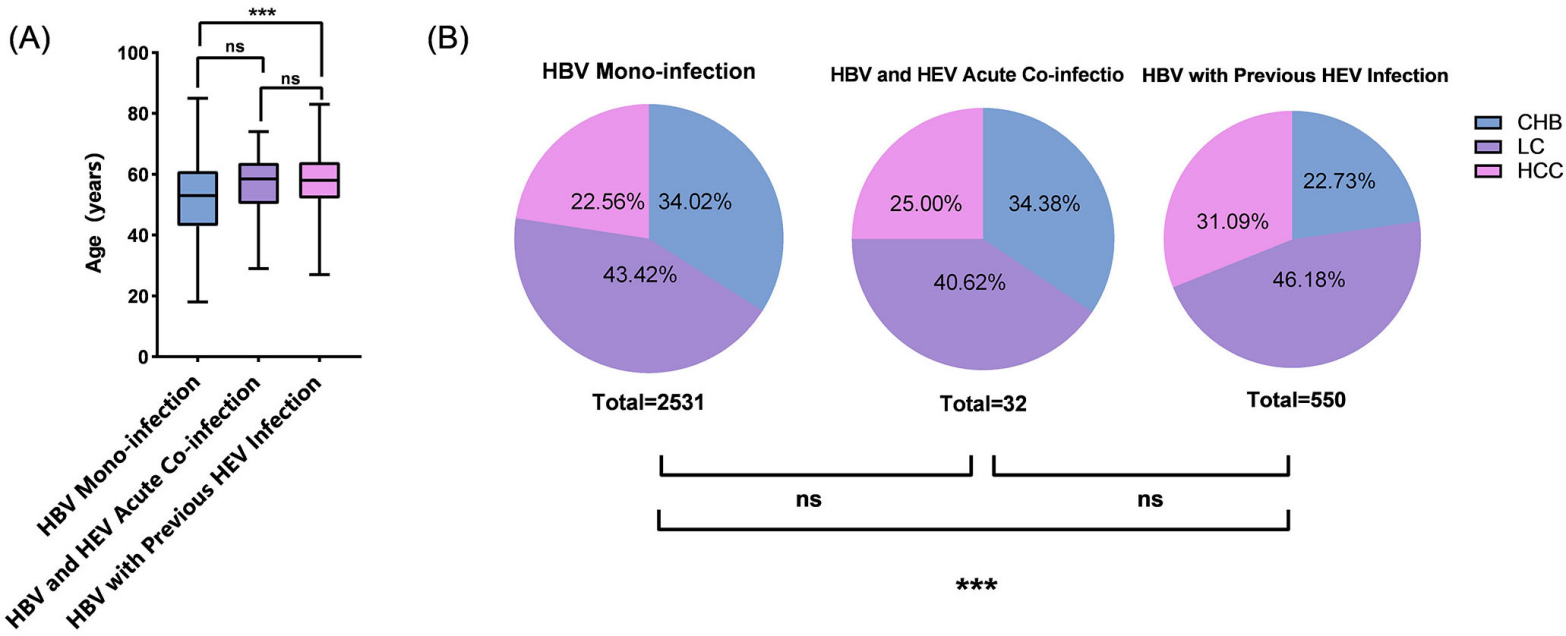
Age distribution and clinical diagnosis composition ratios of the patients in the three groups. **(A)** Age distribution across three groups. Analysis: Kruskal–Wallis test with *post hoc* Mann–Whitney *U* tests. **(B)** Proportional distribution of clinical diagnoses. Analysis: Pearson’s χ^2^ test. Statistical annotations: ns = not significant (*P* > 0.05); ***P < 0.001.

The study population was stratified into three virological profiles: HBV-mono (n = 2,531), HBV/HEV-acute (*n* = 32), and HBV/HEV-previous coinfection (*n* = 550). No significant differences were found in sex distribution or time since onset (*P* > 0.05). However, significant differences were observed in residential areas and occupations (*P* = 0.013 and *P* < 0.001, respectively). The HBV/HEV-acute group had a higher proportion of rural residents compared to both HBV-mono (90.62% vs. 75.03%, *P* < 0.05) and HBV/HEV-previous groups (90.62% vs. 79.45%, *P* < 0.05). The proportion of farmers was significantly higher in the HBV/HEV-previous group than in the HBV-mono group (75.45% vs. 68.71%, *P* < 0.001) (Table 4). HBV-mono patients were younger than HBV/HEV-previous patients (median age: 53.0 vs. 58.0 years; *P* < 0.001). Furthermore, HBV-mono patients had a lower prevalence of LC (43.42% vs. 46.18%) and HCC (22.56% vs. 31.09%) than HBV/HEV-previous patients (*P* < 0.001) (Table 4 and Figure 3).

**TABLE 4:** Demographic characteristics of the patients with the three infection statuses.

Classification	HBV mono-infection (*N* = 2,531)	HBV and HEV acute co-infection (*N* = 32)	HBV with previous HEV infection (*N* = 550)	*P*
Sex, *n* (%)				0.923^a^
Male	1,765 (69.74)	22 (68.75)	388 (70.55)	
Female	766 (30.26)	10 (31.25	162 (29.45)	
Age (years)	53.00 (43.00, 61.00)	58.50 (50.75, 63.25)	58.00 (52.00, 64.00)	<0.001
**Family history, *n* (%)**				
No	1,709 (67.52)	24 (75.00)	375 (68.18)	0.646^a^
Yes	822 (32.48)	8 (25.00)	18 (31.92)	
**Time since onset, *n* (%)**				
0–0.5 month	392 (15.49)	3 (9.38)	72 (13.09)	0.171^b^
0.6–5 year	527 (20.82)	5 (15.62)	124 (22.55)	
6–15 year	878 (34.69)	13 (40.62)	187 (34.00)	
16–25 year	492 (19.44)	7 (21.88)	94 (17.09)	
>25 year	242 (9.56)	4 (12.50)	73 (13.27)	
**Diagnosis, *n* (%)**				
CHB	861 (34.02)	11 (34.38)	125 (22.73)	<0.001^a^
LC	1,099 (43.42)	13 (40.62)	254 (46.18)	
HCC	571 (22.56)	8 (25.00)	171 (31.09)	
Residence area				0.013^b^
Rural	1,899 (75.03)	29 (90.62)	437 (79.45)	
Urban	632 (24.97)	3 (9.38)	113 (20.55)	
**Occupation**				
Farmer	1,739 (68.71)	24 (75.00)	415 (75.45)	<0.001^b^
Worker	201 (7.94)	0 (0.00)	19 (3.45)	
Employee	325 (12.84)	7 (21.88)	74 (13.45)	
Others	266 (10.51)	1 (3.12)	42 (7.64)	
**Antiviral history, *n* (%)**				
No	1,210 (47.81)	11 (34.38)	227 (41.27)	0.008^a^
Yes	1,321 (52.19)	21 (65.62)	323 (58.73)	

^a^Used the Chi-square test.

^b^Used the Fisher-Freeman-Halton exact test and quantitative data were analyzed using the Mann–Whitney *U* test.

### 3.6 Laboratory test results in patients with different HBV/HEV coinfection states

Comparative analysis of hematological and hepatic parameters among HBV-mono, HBV/HEV-acute, and HBV/HEV-previous cohorts revealed significant intergroup variations (omnibus *P* < 0.05) in six key biomarkers: PLT, ALT, PT, INR, PTA, and APTT. Critical findings from Bonferroni-adjusted comparisons revealed two acute coinfection signatures. APTT was significantly longer in HBV/HEV-acute patients than in those with HBV-mono (*P* < 0.05). Compared with HBV-mono patients, HBV/HEV-previous patients had significantly lower PLT, ALT, and PTA levels (*P* < 0.05) and significantly higher PT, APTT, and INR levels (*P* < 0.05). Table 5 presents the results.

**TABLE 5:** Laboratory test results in patients with three infection statuses.

Classification	HBV mono-infection (*N* = 2,531)	HBV and HEV acute co-infection (*N* = 32)	HBV with previous HEV infection (*N* = 550)	*U*	*P*
**Routine blood test**					
WBC (10 × 9/L)	4.55 (3.40, 6.05)	4.65 (3.03, 5.62)	4.50 (3.30, 5.98)	19.3	0.574
Hb (g/L)	134.00 (113.00, 150.00)	131.00 (114.75, 144.75)	131.00 (112.00, 148.00)	4.51	0.170
PLT (10 × 9/L)	131.00 (76.00, 193.00)	123.00 (66.75, 159.00)	117.50 (65.25, 177.75)	0.68	0.002
**Liver function**					
ALT (U/L)	34.00 (21.13, 69.65)	24.40 (17.87, 85.25)	29.70 (20.00, 48.00)	0.07	<0.001
AST (U/L)	34.20 (23.00, 66.50)	28.40 (18.37, 127.50)	32.30 (22.27, 56.75)	2.43	0.105
GGT (U/L)	38.00 (21.00, 85.25)	35.28 (19.23, 101.90)	37.45 (21.94, 81.15)	0.13	0.964
ALB (g/L)	39.60 (33.60, 43.90)	39.20 (36.35, 43.38)	38.80 (32.82, 43.30)	−2.52	0.297
TBIL (μmol/L)	20.60 (13.70, 34.90)	23.74 (15.58, 48.45)	21.61 (14.41, 38.50)	−0.68	0.106
DBIL (μmol/L)	7.10 (4.30, 14.20)	7.90 (5.29, 19.20)	7.15 (4.33, 15.28)	−0.37	0.455
**Coagulation**					
PT (S)	13.50 (11.90, 15.11)	14.10 (12.57, 15.20)	13.80 (12.40, 15.60)	13.64	0.001
INR	1.10 (1.00, 1.26)	1.16 (1.04, 1.26)	1.14 (1.03, 1.28)	10.74	0.005
PTA (%)	85.20 (71.93, 97.54)	80.27 (71.22, 93.08)	82.59 (71.00, 93.54)	10.46	0.005
APTT (S)	32.30 (26.90, 38.70)	35.65 (29.88, 41.50)	34.46 (28.12, 40.69)	22.46	<0.001
Fib (g/L)	2.44 (2.00, 3.04)	2.60 (2.02, 3.00)	2.51 (2.05, 3.07)	2.75	0.253
TT (S)	17.60 (16.40, 18.90)	17.25 (15.90, 18.25)	17.30 (16.30, 18.70)	5.05	0.080

All quantitative data were analyzed using the Mann–Whitney *U* test.

### 3.7 Serological profiling of HBV/HEV replication dynamics in different coinfection states

**FIGURE 4:**
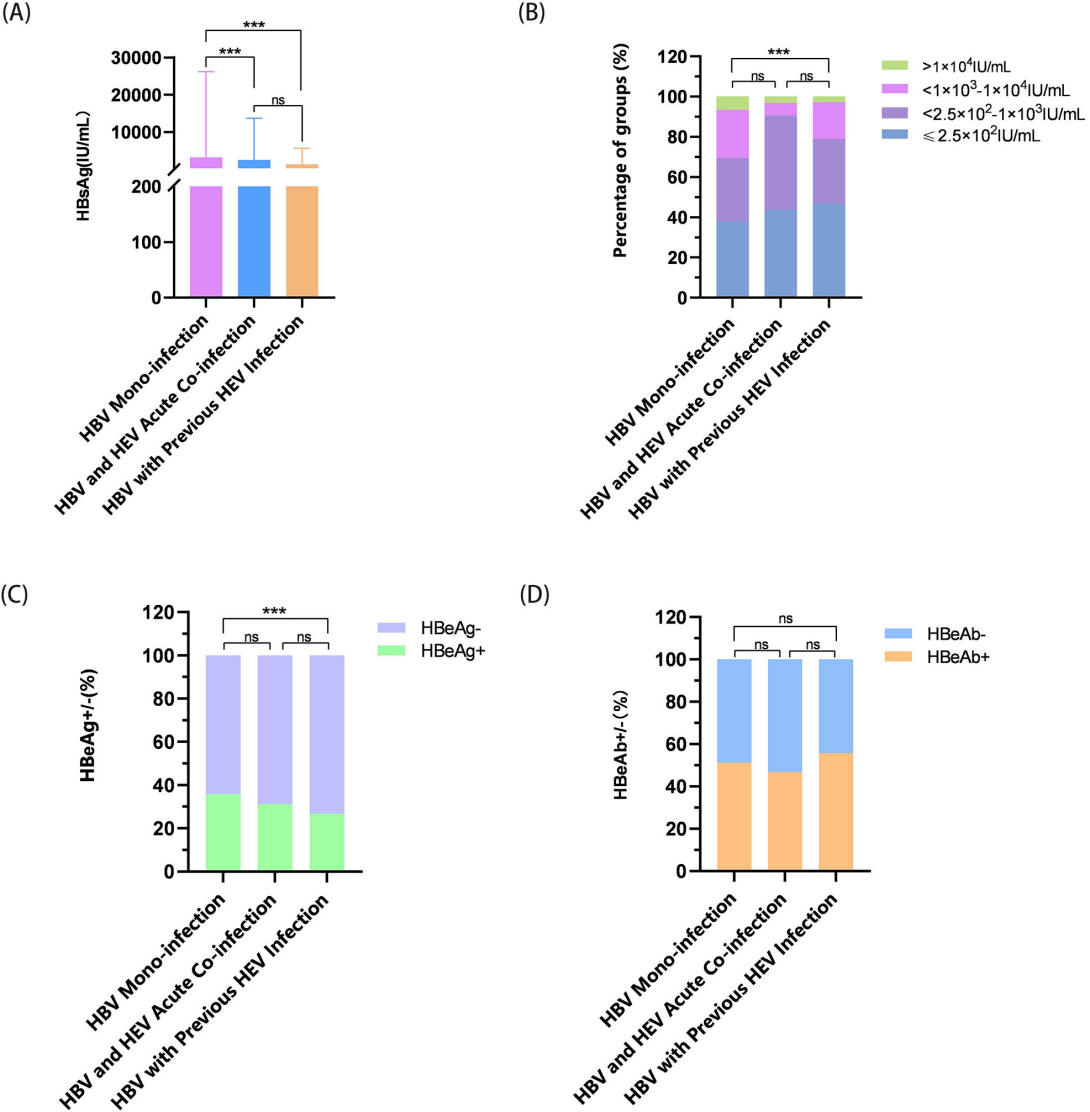
Characteristics of HBsAg levels, and HBeAg and HBeAb positivity rates in the three groups. **(A)** Distribution of serum HBsAg levels across three groups. Analysis: Kruskal–Wallis test with *post hoc* Mann–Whitney *U* tests. **(B)** Proportional distribution of HBsAg quantification subgroups. **(C)** HBeAg-positive rates (%) across three groups. **(D)** HBeAb-positive rates (%) across three groups. Analysis: panels **(B–D)** were analyzed using Pearson’s χ^2^ test. Significance: ns = not significant (*P* > 0.05); ***P < 0.001.

Comparative virological profiling showed significant differences in HBV markers across infection states (*P* < 0.001). HBV-mono patients had higher median HBsAg titers (275.12 IU/ml) than both HBV/HEV-acute (250.1 IU/ml, *P* < 0.001) and HBV/HEV-previous (250.1 IU/ml) patients (Figure 4A). HBV-mono patients also had 2.4 times higher prevalence of high viral loads (>1 × 10^4^ IU/ml) compared to HBV/HEV-previous patients (6.61% vs. 2.76%, *P* < 0.001) (Figure 4B). The HBeAg positivity rate was higher in HBV-mono patients than in HBV/HEV-previous patients (35.95% vs. 26.73%, *P* < 0.05) (Figure 4C). No significant difference was found in HBeAb positivity across groups (*P* > 0.05) (Figure 4D).

**FIGURE 5:**
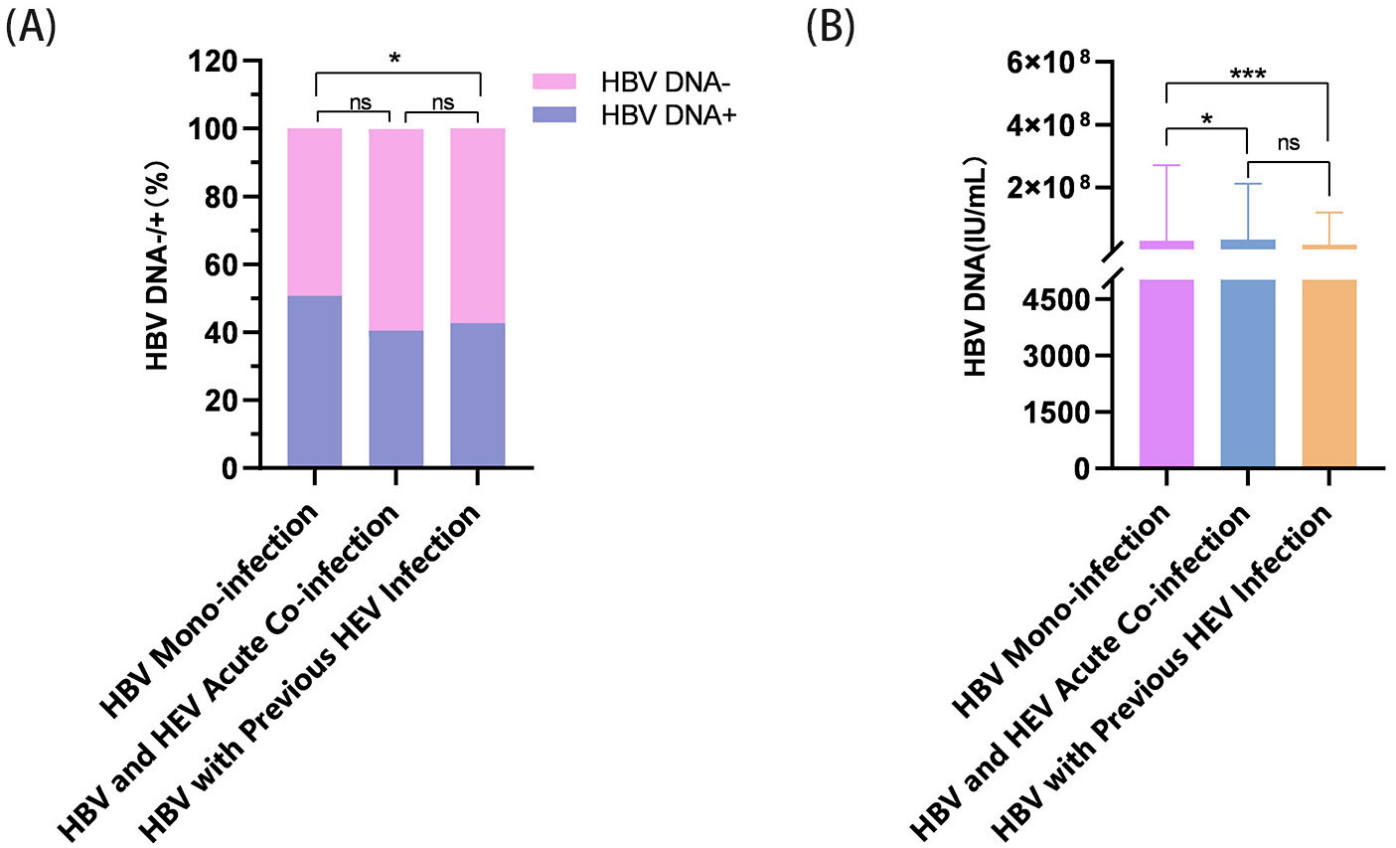
Analysis of serum HBV DNA levels in the three patient groups. **(A)** Serum HBV DNA-positive rates across groups. Analysis: Pearson’s χ^2^ test. **(B)** Proportional distribution of serum HBV DNA level subgroups. Analysis: Kruskal–Wallis test with *post hoc* Mann–Whitney *U* tests. Statistical annotations: ns = not significant (*P* > 0.05); *P < 0.05; ***P < 0.001.

Significant differences were observed in HBV DNA positivity (χ^2^ = 12.56, *P* = 0.002) and DNA load (χ^2^ = 27.00, *P* < 0.001). HBV-mono patients had higher HBV DNA positivity (50.63%) and serum HBV DNA levels compared to the coinfection groups (*P* < 0.001) (Figures 5A, B).

## 4 Discussion

Hepatitis E virus, primarily transmitted via the fecal-oral route, typically causes acute hepatitis but may lead to chronic infection or severe complications in high-risk populations, including immunocompromised individuals and males over 40 years of age (Hoan et al., 2015).

In this study, we observed anti-HEV IgM and IgG positivity rates of 0.64% and 18.57%, respectively, among 3,113 HBV-infected patients. Furthermore, HEV RNA detection rates were significantly higher in IgM+/IgG+ and IgM+ patients than in IgG+ patients (*P* < 0.05), consistent with IgM indicating acute infection and IgG reflecting prior exposure. These findings underscore the clinical utility of combined HEV RNA and IgM testing for accurate diagnosis (Chinese Society of Hepatology, and Chinese Medical Association, 2022). The incidence of HEV infection has increased in recent years (Verghese and Robinson, 2014). Here, the total anti-HEV seroprevalence (18.70%) exceeded previously reported HEV coinfection rates in patients with CHB (2.8%–17.6%) (Nasir and Wu, 2020) and was substantially higher than recent observations in Hebei’s student population (IgG+: 3.4%, IgM+/IgG+: 0.2%) in 2024 and the occupational (IgG+: 13.3%, IgM+: 0.67%) population (Liu et al., 2024). In this study, the prevalence of HBV/HEV coinfection was significantly higher than that of HEV mono-infection. This elevated coinfection prevalence aligns with a Chinese meta-analysis reporting HEV coinfection in CHB patients (Ying-Ying et al., 2020), although regional variations exist. Vietnam has reported higher rates (IgG+: 45%, IgM+: 12%) (Hoan et al., 2015) compared to Turkey/the United States (IgG+: 14%/8%) (Atiq et al., 2009), likely reflecting differences in sanitation and viral genotype distribution. In Asia, HEV genotypes 1 and 4 are predominant (Rizzetto et al., 1977). HEV-4 is the predominant genotype in Jiangsu Province, China (Huang et al., 2024). The genotype in Wuhan, China, is HEV-4. In Zhejiang Province, HEV-4 is also dominant (Chen et al., 2024). Recent studies have shown that HEV-4 is becoming a significant disease burden in immunocompromised individuals, patients with chronic liver disease, and the elderly (Thakur et al., 2020). This study observed the highest prevalence of HEV-IgG in Zhangjiakou, a city located in the agricultural and pastoral regions of northern Hebei Province. This area is characterized by advanced animal husbandry practices, particularly in rural communities with a long-standing tradition of raising livestock such as pigs and rabbits (Geng et al., 2019; Rui et al., 2020; Zhang et al., 2023). In regions with intensive animal husbandry operations, local populations face a significantly elevated risk of HEV exposure through direct contact with animal feces, involvement in slaughtering processes, and consumption of undercooked animal offals (Kuniholm et al., 2009).

Through a multifactorial analysis, this study confirmed that advancing age is an independent risk factor for anti-HEV seropositivity. Furthermore, the mean age of patients with HBV with previous HEV coinfection was significantly higher than that of HBV mono-infection patients. Analysis stratified by clinical diagnosis revealed a significant age-dependent increase in anti-HEV seropositivity in patients with LC and HCC. Conversely, patients with CHB exhibited only a gradual increase in positivity. Specifically, in the >55 years age group, the anti-HEV seropositivity rates were significantly higher in the LC and HCC groups than in the CHB group. A study in Jiangsu Province indicates that individuals aged 50–69 have a higher incidence of HEV. In general, the elderly are more susceptible to hepatitis E due to a decline in immune function (Huang et al., 2024). This observation may be partially explained by the natural decline in immune function associated with aging (Fan et al., 2020; Hoan et al., 2015). Serological investigations conducted in the Netherlands also confirmed an age-related increase in HEV antibody positivity, consistent with cumulative exposure over time and birth cohort effects. These findings collectively reinforce the hypothesis of an exposure accumulation mechanism (van Gageldonk-Lafeber et al., 2017), in which prolonged environmental or occupational contact with HEV reservoirs may contribute to higher seroprevalence in older populations. In a study in Zhejiang Province, HEV infection was found to be associated not only with age but also with frequent dining out, poor hygiene habits, and unhealthy behaviors (Chen et al., 2024). Although the results of this study provide insight into the relationship between HEV infection and age in the population of Hebei Province, we also acknowledge that the genotype of HEV, environmental factors, and region-specific risk factors may influence the results.

The results of our analyses indicated that the risk of HEV positivity was significantly higher in the farmer group than in the other groups (e.g., students, self-employed workers, and retirees). HEV is primarily transmitted through food. Farmers are typically exposed to zoonotic pathogens over extended periods, particularly when handling livestock or interacting with irrigation systems, which increases the risk of infection (Mrzljak et al., 2021). Studies conducted in China have also reported higher IgG seropositivity rates among farmers (Kang et al., 2017; Yue et al., 2019). Conversely, workers generally operate in environments with more robust protective measures such as overalls, protective equipment, and proper sanitation, which effectively reduce the risk of infection (Liu et al., 2024). Additionally, our research revealed that the proportion of rural populations in the HBV/HEV-acute group was significantly higher than that in the HBV-mono and HBV/HEV-previous groups. This suggests that acute HEV infection may be more easily transmitted in rural areas, possibly because of the higher risk of exposure to contaminated water sources or animal hosts. Previous studies have pointed out that geographic environment and living conditions may play a role in the development of HEV infection. For example, the overall seroprevalence of anti-HEV IgG in rural areas (28.7%) was higher than that in urban areas (21.7%), with an odds ratio of 2.17 (95% CI: 1.52–3.11), underscoring the importance of environmental and occupational factors in HEV transmission (Toole et al., 2006).

Our findings demonstrated that both LC and HCC were more prevalent in HBV patients with previous HEV infection than in HBV mono-infection patients, and the anti-HEV seropositivity rate was substantially higher in LC and HCC patients. Previous studies have shown that the prevalence of HEV infection is markedly higher in LC patients (3.3%) than in non-LC populations (0.2%–2%), with coinfection exacerbating hepatic injury and significantly increasing the risk of liver failure and associated mortality (Tseng et al., 2020). Other studies have confirmed a positive association between HBV/HEV coinfection and an elevated HCC incidence (Xue et al., 2021; Yang et al., 2019). We observed significant differences in PLT and ALT levels between HBV/HEV-previous and HBV-mono infected groups. The decreased PLT and ALT, along with elevated PT and APTT, suggest more severe liver injury in coinfected patients, especially those with LC and HCC. This highlights the need for closer monitoring of liver function and coagulation parameters, and potentially more aggressive treatment strategies. Mechanistically, dual HEV infection may exacerbate hepatocellular damage by interfering with the normal cytokine secretion pathways (Kilonzo et al., 2019). Additionally, prolonged PT/APTT and elevated INR in HBV/HEV coinfected patients suggest coagulation impairment, which may stem from acute endothelial injury, protein consumption, and underlying liver dysfunction (Izopet et al., 2017). These findings suggest that clinicians should consider routine HEV screening in HBV patients, particularly those with pre-existing liver damage such as LC and HCC. Additionally, this highlights the need for frequent coagulation monitoring and the potential necessity for more aggressive therapeutic strategies to manage bleeding risks.

Interestingly, our findings revealed that HBV mono-infection patients exhibited higher titers of HBsAg, HBeAg seropositivity rates, and HBV DNA loads than the HBV/HEV coinfection groups. Research has shown that compared to HBV patients without HEV infection, those with a history of HEV infection have lower HBV-DNA levels, indicating that the host immune response helps control HBV replication (Hoan et al., 2015). Wang (2015) reported lower HBV DNA levels in HEV/HBV co-infected patients, while Hoan et al. (2015) observed reduced HBV DNA levels in individuals with current HEV infection compared to those with a history of HEV infection. Cheng et al. (2013) provided further evidence that acute HEV infection could induce HBV to enter a dormant state. Additionally, a case report documented HBsAg clearance during acute HEV exacerbation (Yeh et al., 2018). However, this phenomenon must be interpreted in the context of the natural history of HBV infection. Patients with LC and HCC typically have a prolonged disease course during which long-term interactions between the immune system and HBV may lead to the virus entering a low-replicative state. As a result, their serum HBsAg and HBV DNA loads were generally lower than those of patients with CHB. Although several studies have shown that HEV coinfection may suppress HBV replication, the findings across the literature remain inconsistent. A Vietnamese cohort study reported a 1.7-fold increase in HBV DNA load among HEV-coinfected individuals (Hoan et al., 2015), whereas Kumar Acharya et al. (2007) found no significant association between these two viral infections. This complex interplay likely involves multiple factors, necessitating further investigations that integrate viral genotyping and analyses of host immune characteristics to elucidate the underlying mechanisms.

This province-wide study revealed a substantial burden of HEV coinfection among patients with chronic HBV in Hebei, China, with 18.70% anti-HEV seroprevalence (IgG+: 18.57%, IgM+: 0.64%) and 0.51% active viremia (HEV RNA+). The risk of HEV infection increases significantly with age. The relatively high proportions of LC and HCC in anti-HEV-positive patients suggest that HEV infection may exacerbate liver injury, potentially affecting coagulation and hepatic metabolic functions.

This study provides the first systematic characterization of HBV/HEV coinfection patterns in northern China. Our findings underscore the urgent need for integrated HEV screening in HBV management protocols, particularly among rural populations, the elderly, and patients with advanced liver disease, where such screening could lead to earlier detection and improved management. Targeted screening in these high-risk groups could help reduce the burden of liver injury and improve clinical outcomes. Future research should focus on longitudinal analyses of viral kinetics, genotype-specific virulence assessments, and immune response dynamics in coinfected patients. Additionally, investigating the impact of occupational and environmental exposures on HEV transmission could further guide public health strategies and optimize therapeutic interventions.

### 4.1 Limitations

This study has several limitations. Its cross-sectional design precludes causal inference between HEV infection and disease progression. Key exposure factors (e.g., diet, water source, and occupation) were unavailable due to limited medical record documentation. The small HBV/HEV acute cohort (*n* = 32) restricted acute infection analysis, and missing genotype data precluded subtype-specific insights. Moreover, focusing on hospitalized HBsAg-positive patients may introduce selection bias, underrepresenting asymptomatic and early-stage cases and potentially overestimating HEV prevalence. Future studies should collect detailed exposure data, include broader populations, and adopt longitudinal, multicenter designs with genotyping to better define HEV’s burden across the HBV spectrum.

## Data Availability

The raw data supporting the conclusions of this article will be made available by the authors, without undue reservation.
